# Validation of a short Italian version of the Barratt Impulsiveness Scale (BIS-15) in non-clinical subjects: psychometric properties and normative data

**DOI:** 10.1007/s10072-022-06047-2

**Published:** 2022-04-11

**Authors:** Gianpaolo Maggi, Manuela Altieri, Ciro Rosario Ilardi, Gabriella Santangelo

**Affiliations:** Department of Psychology, University of Campania “Luigi Vanvitelli”, Caserta, Italy

**Keywords:** Impulsivity, Barratt Impulsiveness Scale, Psychometric properties, Normative data

## Abstract

**Introduction:**

The Barratt Impulsiveness Scale (BIS) is a questionnaire employed to measure impulsivity, which is associated with risky behaviors and mental disorders. We aimed to assess the psychometric properties of the BIS in the Italian general population and provide normative data for clinical use.

**Materials and methods:**

A cross-sectional survey methodology was employed to collect data. Then, 534 participants of different ages and educational levels completed the BIS, PHQ-9, GAD-7, and S-UPPS-P. We designed an ad hoc data-driven outcome checklist to identify which items deserved to be retained. Furthermore, internal consistency, convergent and divergent validity, and factorial structure were evaluated. A regression-based procedure was used to explore the influence of demographic variables on the BIS score and to provide adjusting factors and a sensitive cutoff.

**Results:**

Item analysis suggested removing 15 items. Consequently, we tested the psychometric properties of a shorter version of the BIS (BIS-15). IRT test information functions indicated an almost identical measurement precision of the BIS-15 as compared to the original BIS. The BIS-15 demonstrated reliable internal consistency and good convergent and divergent validity. The PCA revealed a four-factor solution: “pure impulsivity,” “planning and thinking,” “lack of attention and concentration,” and “impulsive buying.” A significant effect of sex and years of education was found. Norms for the adjustment of raw scores were provided (cutoff = 37.39).

**Conclusions:**

The BIS-15 showed almost identical psychometric properties as compared with the original scale, reducing the administration time. Our norms may allow identifying individuals with impulsivity of clinical interest.

**Supplementary Information:**

The online version contains supplementary material available at 10.1007/s10072-022-06047-2.

## Introduction

Impulsivity (or impulsiveness) is an important psychological construct comprising a heterogeneous cluster of traits such as impulsiveness, novelty-seeking, risk-taking, boredom susceptibility, sensation and excitement seeking, untidiness, unreliability, and monotony avoidance [1]. Since there is high interindividual (some people are more impulsive than others) and intraindividual (in some occasions or life stages, individuals behave more impulsively) variability in impulsive behaviors during the lifetime, the construct is interpreted to reflect a continuum of a personality features or traits.

From a clinical standpoint, the DSM-5 [2] defined impulsivity as an immediate and unplanned reaction to stimuli on the spur of the moment or without considering its consequences. Moreover, it appears in the diagnostic criteria for several psychiatric disorders characterized by risky behaviors such as drug addiction, bipolar disorder, eating disorders, personality disorders, and attention-deficit/hyperactivity disorder [3]. Furthermore, the occurrence of impulsivity has been detected in several neurological diseases. For instance, impulse control disorders are commonly observed in Parkinson’s disease [4] with a negative impact on patients’ cognitive abilities [5]; moreover, patients with multiple sclerosis show higher motor and cognitive impulsivity as compared with healthy subjects [6], and increased impulsivity levels are reported in patients with amyotrophic lateral sclerosis [7].

Taking into account the frequent occurrence of impulsivity in several psychiatric and neurological diseases, its detection becomes relevant not only in terms of diagnostic accuracy but also for promptly providing adequate treatments.

Several scales were developed to assess impulsiveness in healthy subjects and clinical populations. Out of these, the Barratt Impulsiveness Scale (BIS), a 30-item questionnaire, represents the most commonly administered self-report measure specifically designed to assess the personality/behavioral construct of impulsiveness in both research and clinical settings [8].

The latest version of the BIS, the BIS-11 by Patton and colleagues [9], was characterized by six first-order latent factors such as attention, motor, self-control, cognitive complexity, perseverance, and cognitive instability and three second-order factors labeled as attentional, motor, and nonplanning impulsiveness, respectively. This factorial structure supports the idea of impulsiveness as a multi-dimensional construct.

The Italian translation and psychometric properties of the Patton’s BIS-11 were provided by Fossati and colleagues [10] who administered the scale to college undergraduate students. Moreover, an adolescent Italian version of the BIS-11 was also developed and standardized [11]. However, no study provided normative data and psychometric properties of the BIS in the Italian general population.

Therefore, the present study aimed to explore the psychometric properties of the BIS (i.e., reliability, information, convergent validity, divergent validity, and factorial structure) and extract normative values for the Italian general population.

## Materials and methods

### Participants

Participants were recruited through a cross-sectional survey methodology employing an online questionnaire using Google Forms platform. Participation in the survey was open from April 20 to June 24, 2021. Completing the questionnaire took approximately 20 min.

The questionnaire was disseminated to university students, friends, colleagues, and acquaintances via a snowball sampling strategy but also in virtual environments (i.e., Facebook, Whatsapp, and social virtual groups) to recruit a large Italian sample of people living in different Italian regions.

The survey included several sections: (1) socio-demographic information; (2) health status questionnaire; (3) the Italian version of the BIS-11, translated by Fossati and colleagues [10]; (4) the Positive Urgency subscale of the Italian short form of the UPPS-P Impulsive Behavior Scale (S-UPPS-P) for assessing convergent validity; (5) the 9-item Patient Health Questionnaire-9 (PHQ-9) and the 7-item Generalized Anxiety Disorder scale (GAD-7) for evaluating divergent validity.

Socio-demographic information included data about sex, age, and years of formal education according to the Italian schooling system.

The health status questionnaire was useful to identify subjects suffering from previous and/or current psychiatric and/or neurological disorders (i.e., epilepsy, stroke, multiple sclerosis, migraine, cognitive impairment, eating disorders, major depressive disorder, anxiety, panic attacks) and people under treatment with antidepressants and/or other mental health medications. In order to provide Italian normative values of the BIS-11, all subjects with any neurological or psychiatric disorders were excluded.

The BIS-11 is a self-report questionnaire designed to measure impulsiveness [12] and consists of 30 items with a 4-point Likert scale ranging from 1 (never/rarely) to 4 (almost always/always); however, some items present a reverse coding to avoid response bias. Higher scores on the BIS-11 indicated a higher level of impulsiveness.

The Positive Urgency subscale of the Italian short form of the UPPS-P Impulsive Behavior Scale (S-UPPS-P) assesses the tendency to act rashly under extreme positive emotions through 20 items answered on a 4-point Likert scale.

The 9-item Patient Health Questionnaire-9 (PHQ-9) assesses the DSM-5 symptoms for a major depressive episode, while the 7-item Generalized Anxiety Disorder scale (GAD-7) explores symptoms for Generalized Anxiety Disorder as defined by the DSM-IV.

All participants gave their informed consent before completing the online questionnaire. The present study has been approved by the Local Ethics Committee and performed in accordance with the ethical standards laid down in the 1964 Declaration of Helsinki together with the principles that guide the ethical and methodological practice of online research [13, 14].

### Statistical analysis

Quality of normative data was evaluated by the percentage of missing values and the presence of floor and ceiling effects. Percentages of < 5% of missing values and < 15% of the respondents with the lowest or highest scores (floor and ceiling effects) are considered indices of optimal data quality [15]. Univariate normality was assessed by checking skewness and kurtosis values. Values ranging between − 2 and + 2 are typically indicative of no significant distortions from the Gaussian distribution [16]. The scale’s internal consistency was evaluated by Cronbach’s alpha coefficient, with a value ≥ 0.70 that was considered acceptable*.* For each item, additional evidence on the reliability and scaling assumptions was obtained by Pearson’s item-total correlations and corrected item-total correlations to adjust inflation errors. As for the former, Cohen’s conventions (weak, *r* < 0.30; moderate, *r* = 0.30–0.50; strong, *r* > 0.50) [17] were used to interpret the effect size; as for the latter, a value > 0.30 was deemed acceptable in terms of both consistency and discriminative capability [18, 19]. Furthermore, item response theory (IRT) analysis for polytomous items was conducted to quantify the amount of psychometric information in the single items, and the scale as a whole, provided for each level of the latent trait (*θ*), i.e., impulsivity. Information represents the measurement precision and thus it is inversely related to the standard error. Higher information is associated with less measurement errors and more reliable estimates of *θ* [20, 21].

To determine which items deserved to be included in the Italian version of the BIS, a systematic data-driven approach involving the compilation of an ad hoc checklist was applied (see Supplementary Material 1). Specifically, inclusion criteria were at least (i) a weak-to-moderate correlation with the total BIS score, (ii) an acceptable corrected item-total correlation, and (iii) a portion of the *θ* continuum capturing information values > 0.2 according to IRT analysis. An item was dropped if it failed to satisfy at least two out of the three above criteria.

Principal component analysis (PCA) with Varimax orthogonal rotation was used to evaluate the factorial structure of the scale. The Mineigen criterion (eigenvalues > 1 [22]) and the inspection of the scree plot were employed to determine the number of factors to be extracted [23].

Convergent validity was assessed by Pearson’s correlation between BIS and S-UPPS-P Positive Urgency score. Divergent validity was assessed by correlation between the BIS total score and the scores of the PHQ-9 and GAD-7.

To evaluate the potential influence of demographic factors (i.e., age, educational level, and sex) on the BIS total score, linear regression analyses were performed. At first, the effects of continuous demographical variables were tested by simple regression analyses after several mathematical transformations (e.g., square root, logarithmic) to determine the most effective in reducing the residual variance of the BIS total score. Then, we carried out a multiple regression analysis entering the BIS total score as dependent variable and age, education level, and sex as independent variables. Subsequently, the best fitting simultaneous linear regression model was constructed to adjust the raw BIS score based on the significant sociodemographic variables. Bonferroni’s correction for reducing the inflation of the type I error was applied; therefore, the significance threshold was fixed at 0.05/*k*, where 0.05 is the nominal alpha level and *k* is the number of independent variables (*p* = 0.0167).

Finally, we calculated the adjusting factors by adding or subtracting the contribution of significant predictors from the raw scores. A non-parametric procedure, with a set of confidence at 95%, was applied to estimate the cutoff, which was fixed at the inner tolerance limit of the 95th centile of the adjusted distribution. Statistical analyses were performed with IBM SPSS Statistics (v. 26) and STATA (v. 15) packages.

## Results

A total number of 648 participants completed the online questionnaire; however, 114 participants did not meet all the inclusion criteria (i.e., they reported the presence of a neurological/psychiatric disorder, cognitive decline, or ongoing treatment with psychotropic drugs) and were excluded from the analysis. The final sample consisted of 534 participants (152 males and 382 females) with a mean age of 36.82 (*SD* = 13.65) and an average education of 15.29 years (*SD* = 2.52). The distribution of the sample for age and education is reported in Table 1.

**Table 1:** Normative sample stratified by age, sex, and education (*N* = 534)

	Age, years					
	18–30	31–40	41–50	51–60	61 +	Total
Low education (0–13 years)						
Males	35	14	8	11	7	75
Females	58	37	23	21	11	150
High education (> 13 years)						
Males	33	14	12	9	9	77
Females	114	55	32	21	10	232
Total						
Males	68	28	20	20	16	152
Females	172	92	55	42	21	382

No significant differences were found between males and females on age (*F*(1, 532) = 10.151, *p* = 0.078) and educational level (*F*(1, 532) = 3.492, *p* = 0.090). The skewness and kurtosis values were in the normality range (− 2.0 and + 2.0) for each variable under examination. As for data acceptability, we did not find any missing data, neither floor nor ceiling effects. The mean BIS-11 score was 58.44 (*SD* = 8.61).

### Reliability

Half of the items of the BIS showed significant moderate (items 1, 4, 5, 8, 10, 12, 13, 17, 18, 22, 25; *r* range = 0.396–0.499, *p*_s_ < 0.001) to strong correlations (items 2, 9, 14, 19; *r* range = 0.500–0.554, *p*_s_ < 0.001) with the total score, and an acceptable level of discrimination (corrected item-total correlations, range = 0.320–0.486; Table 2). Conversely, items 6, 7, 11, 15, 16, 20, 21, 23, 24, and 26–30 showed weak-to-moderate correlations (*r* range = 0.150–0.373) with the total score and unsatisfactory level of discrimination (corrected item-total correlations, range = 0.054–0.289; Table 2). Finally, item 3 was not correlated with the total score (*r* = 0.072, *p* = 0.095) and had a very poor level of discrimination (corrected item-total correlation =  − 0.026; Table 2). As a whole, the scale demonstrated acceptable internal consistency as shown by a Cronbach’s alpha of 0.755.

**Table 2:** Barratt Impulsiveness Scale item characteristics

	Mean ± SD	Item-total correlation	Corrected item-total correlation	Cronbach’s alpha if item removed
1. I plan tasks carefully	2.10 ± 0.84	0.415	0.330	0.745
2. I do things without thinking	1.76 ± 0.71	0.530	0.466	0.739
3. I make up my mind quickly	2.32 ± 0.85	0.072	− 0.026	0.765
4. I am happy-go-lucky	1.62 ± 0.69	0.432	0.364	0.744
5. I don’t “pay attention”	1.75 ± 0.80	0.467	0.390	0.742
6. I have “racing” thoughts	2.81 ± 0.94	0.216	0.108	0.758
7. I plan trips well ahead of time	2.41 ± 1.04	0.179	0.060	0.763
8. I am self-controlled	1.93 ± 0.80	0.475	0.399	0.741
9. I concentrate easily	2.17 ± 0.80	0.500	0.426	0.740
10. I save regularly	2.27 ± 0.91	0.456	0.366	0.742
11. I “squirm” at plays or lectures	1.79 ± 0.94	0.350	0.248	0.750
12. I am a careful thinker	1.94 ± 0.76	0.396	0.318	0.746
13. I plan for job security	2.14 ± 0.90	0.417	0.324	0.745
14. I say things without thinking	1.69 ± 0.73	0.549	0.485	0.738
15. I like to think about complex problems	2.47 ± 0.98	0.168	0.054	0.762
16. I change jobs	1.38 ± 0.62	0.150	0.078	0.757
17. I act “on impulse”	2.01 ± 0.76	0.499	0.428	0.740
18. I get bored easily when solving thought problems	1.79 ± 0.79	0.461	0.385	0.742
19. I act on the spur of the moment	1.96 ± 0.78	0.554	0.486	0.737
20. I am a steady thinker	2.10 ± 0.85	0.297	0.204	0.752
21. I change residences	1.25 ± 0.52	0.199	0.140	0.754
22. I buy things on impulse	1.71 ± 0.73	0.396	0.320	0.746
23. I can only think about one problem at a time	1.82 ± 0.84	0.199	0.103	0.758
24. I change hobbies	1.71 ± 0.77	0.312	0.227	0.751
25. I spend or charge more than I earn	1.40 ± 0.69	0.427	0.358	0.744
26. I often have extraneous thoughts when thinking	1.75 ± 0.80	0.373	0.289	0.747
27. I am more interested in the present than the future	2.15 ± 0.83	0.317	0.226	0.751
28. I am restless at the theater or lectures	1.49 ± 0.77	0.365	0.283	0.748
29. I like puzzles	2.66 ± 0.98	0.231	0.119	0.758
30. I am future oriented	2.09 ± 0.84	0.353	0.264	0.749

### Item and test information

**Fig. 1:**
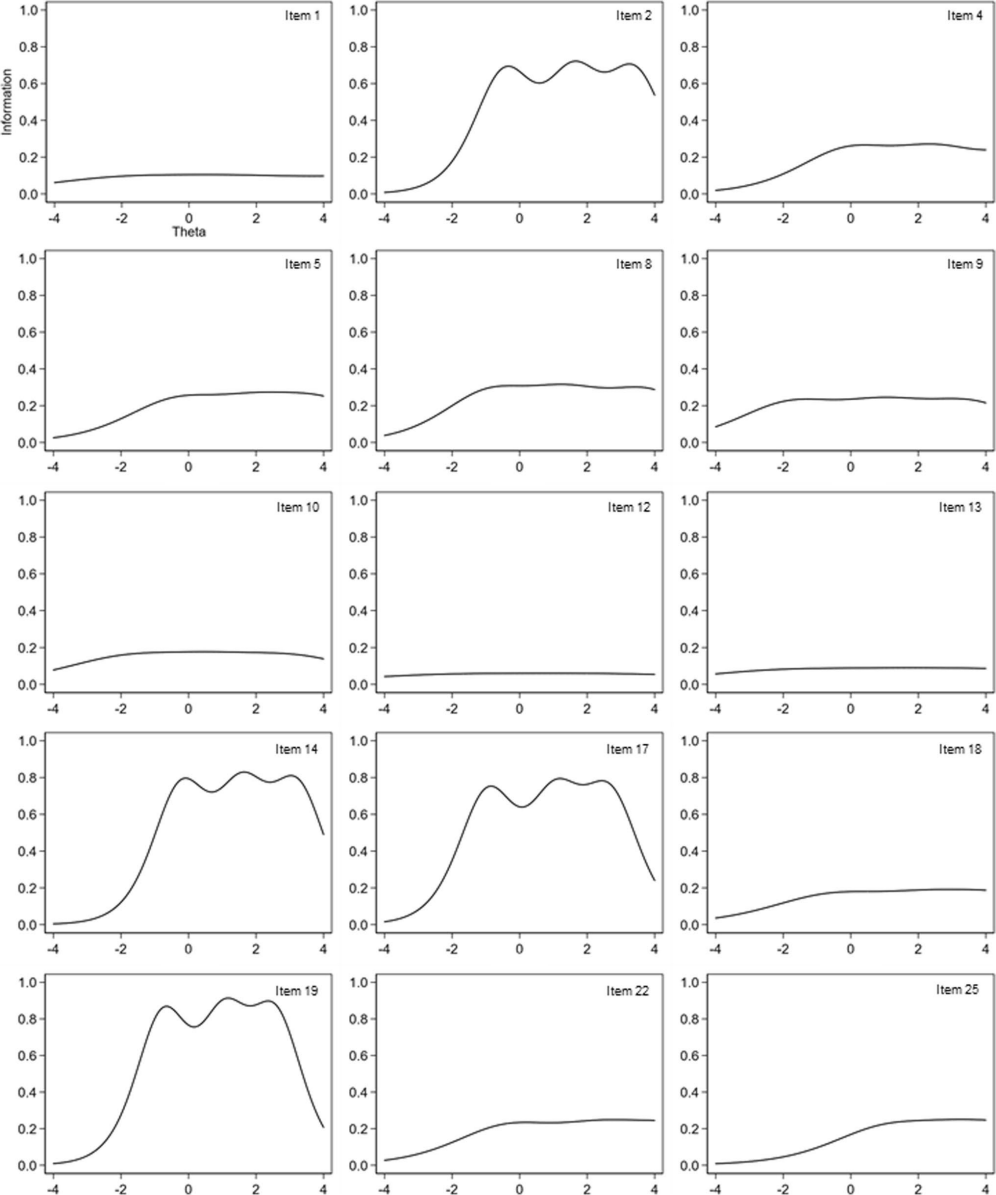
Item information functions for the included items. Note: The *x*-axis (theta) represents the level of impulsiveness on the latent trait continuum, while the *y*-axis, the amount of information (precision) available in the item

Three IRT–based models for ordinal items were tested: the Graded Response (GRM), the Partial Credit (PCM), and the Generalized Partial Credit (GPCM) models. To compare the relative fit of these models, the Likelihood Ratio Test (LRT) was used; moreover, the Akaike Information Criterion (AIC) and the Bayesian Information Criterion (BIC) were computed, where smaller AIC and BIC values indicate a better fit. Results of LRT showed that both GRM and GPCM fitted better compared with PCM (PCM < GRM, LR *χ*^*2*^(29) = 599.21, *p* < 0.001; PCM < GPCM, LR *χ*^*2*^(29) = 580.19, *p* < 0.001). The GRM was selected as the best fitting model according to AIC and BIC values (GRM, *AIC* = 34,746.34, *BIC* = 35,259.99; GPCM, *AIC* = 34,765.96, *BIC* = 35,279.60). Based on discrimination and difficulty parameters (see Supplementary Material 2), Item Information Functions (IIFs) were constructed (see Fig. 1 and Supplementary Material 3). Twenty items (i.e., 1, 3, 6, 7, 10–13, 15, 16, 18, 20, 21, 23, 24, 26–30) demonstrated to be not very precise in measuring impulsivity at any location along the latent variable (information provided ≤ 0.2).

### Item selection and scale abbreviation: the BIS-15

Based on the outcome checklist, fifteen items (i.e., 3, 6, 7, 11, 15, 16, 20, 21, 23, 24, 26–30) did not meet the inclusion criteria and were dropped (see Supplementary Material 1). When these items were removed, Cronbach’s alpha increased from 0.755 to 0.793. This finding further confirmed that the above items were not internally consistent and deserved to be excluded. The remaining fifteen items (i.e., 1, 2, 4, 5, 8–10, 12–14, 17–19, 22, 25) will constitute a shortened version of the Italian BIS that, now onwards, will be referred to as “BIS-15” (for the English version and the one translated into the Italian language see Supplementary Material 4).

The IIFs of the included items are displayed in Fig. 1. Among the included items, items 2, 14, 17, and 19 provided the largest amount of information (~ 0.70–0.90), particularly for the ability levels between − 1 and + 3; furthermore, they showed strong correlations with the total BIS score and corrected item-total correlations > 0.40. Items 4, 5, 8, 9, 22, and 25 provided, instead, information values ranging from ~ 0.20 to ~ 0.30 for individuals with *θ* levels between − 2 and + 4; moreover, these items showed moderate-to-strong correlations with the total BIS score as well as corrected item-total correlations > 0.30. Finally, items 1, 10, 12, 13, and 18 provided the lowest amount of information (~ 0.04–0.20). However, their IIFs were almost horizontal, with all ability levels being estimated with the same precision. These items showed moderate correlations with the total BIS score and corrected item-total correlations > 0.30.

**Fig. 2:**
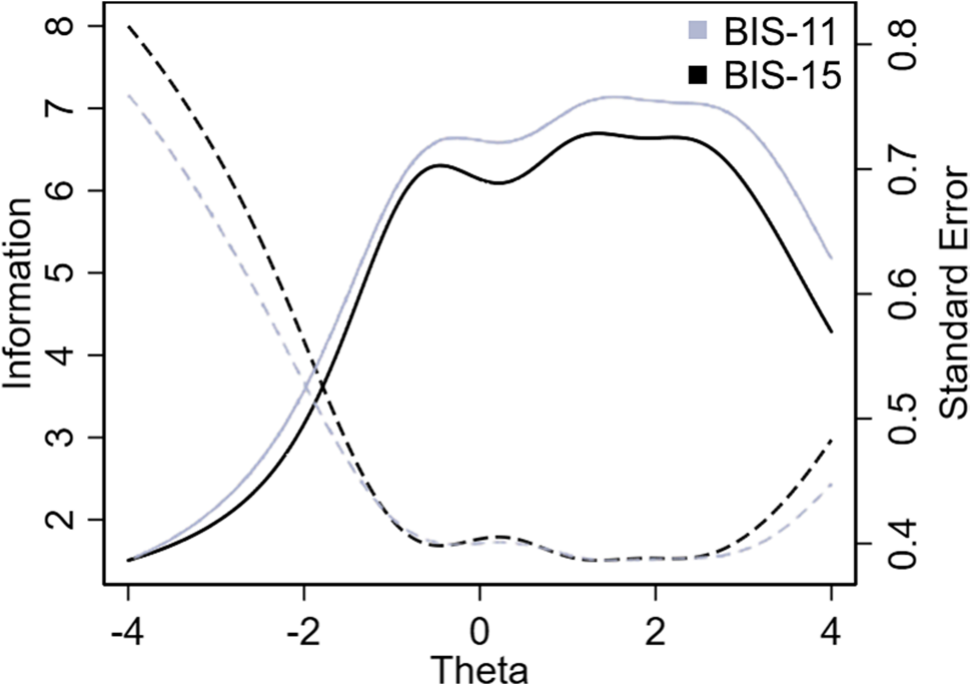
Comparison between test information functions (TIFs) of BIS-11 and BIS-15. Note: Solid lines represent the information curves and dashed lines, the standard errors. The *x*-axis (theta) represents the level of impulsiveness on the latent trait continuum, while the *y*-axis, the amount of information (precision) provided by each scale. The two functions almost overlapped and therefore the loss of information from BIS-11 to BIS 15 was negligible. Both scales reached higher precision levels around the latent trait between − 1 and + 3

As the reliability of the single items is of relative interest if compared to the entire scale, we compared the original and shortened BIS to further verify if the scale’s abbreviation resulted in a significant loss in measurement precision. Accordingly, Test Information Functions (TIFs), before and after items’ removal, were constructed by combining information functions from each item. As shown in Fig. 2, the TIF of the original scale (30 items) and the TIF of the Italian BIS (15 items) were almost identical.

### Factorial analysis of the BIS-15

A four-factor solution was generated by PCA using the Mineigen criterion (eigenvalues > 1; Table 3) and this result was confirmed after the inspection of the scree plot.

**Table 3:** Principal component analysis for the BIS-15

	Factor 1	Factor 2	Factor 3	Factor 4
17. I act “on impulse”	**0.845**	0.003	0.024	0.034
19. I act on the spur of the moment	**0.814**	− 0.008	0.084	0.123
2. I do things without thinking	**0.637**	0.172	0.187	0.110
14. I say things without thinking	**0.635**	0.198	0.212	0.089
4. I am happy-go-lucky	**0.485**	0.042	0.155	0.110
13. I plan for job security	0.099	**0.707**	0.018	0.017
12. I am a careful thinker	− 0.066	**0.637**	0.141	0.197
1. I plan tasks carefully	0.147	**0.610**	0.039	0.051
8. I am self-controlled	0.430	**0.447**	0.120	0.052
5. I don’t “pay attention”	0.177	0.143	**0.761**	0.009
9. I concentrate easily	0.077	0.375	**0.724**	0.010
18. I get bored easily when solving thought problems	0.242	− 0.159	**0.586**	0.158
25. I spend or charge more than I earn	0.143	− 0.044	0.218	**0.795**
10. I save regularly	0.068	0.323	0.024	**0.775**
22. I buy things on impulse	0.424	0.098	− 0.130	**0.504**
Variance explained (%)	26.87	10.80	8.62	7.41
Correlation (*r*) with total score	0.798*	0.708*	0.652*	0.654*
Cronbach’s α	0.776	0.546	0.579	0.599

Major loadings for each item are displayed in bold. *p < 0 .001

The first factor was composed of items that loaded under a *pure impulsivity* factor such as “I act on the spur of the moment” and “I say things without thinking” (explained variance = 26.87%). The second factor included items reflecting a *planning and thinking* factor like “I plan tasks carefully” and “I am a careful thinker” (explained variance = 10.80%). The third factor was composed of items indicating an impulsivity dimension related to the *lack of attention and concentration* such as “I don’t pay attention” and “I concentrate easily” (explained variance = 8.62%). Finally, the fourth factor, *impulsive buying*, included items mainly related to the urge to buy impulsively things as “I spend or charge more than I earn” and “I save regularly” (explained variance = 7.41%).

### Convergent and divergent validity

Convergent validity was assessed by correlating the BIS-15 score with the Positive Urgency of the S-UPPS-P: a significantly strong correlation emerged between the two measures (*r* = 0.514, *p* < 0.001).

Conversely, divergent validity was measured by correlating the BIS-15 score with GAD-7 and PHQ-9. We found a weak but significant correlation with the GAD-7 (*r* = 0.273, *p* < 0.001) and a moderate association with the PHQ-9 (*r* = 0.371, *p* < 0.001).

### Normative data of the BIS-15

Simple linear regression models were designed assuming the BIS-15 score as dependent variable, and age and education (in years) as predictors. Both age and education variables did not need any mathematical transformations since raw scores were found to be the most effective in reducing the residual variance of the BIS-15 score.

Multiple regression analysis entering age, sex, and education as predictors and BIS-15 score as dependent variable revealed a significant effect of sex (*B* = 2.207, *t* = 3.931, *p* < 0.001) and education (*B* =  − 0.374, *t* =  − 3.721, *p* < 0.001) but no effect of age (*B* =  − 0.008, *t* =  − 0.446, *p* = 0.656). Then, we calculated the best-fitting linear model and constructed a correction grid to allow the adjustment of the BIS-15 raw score (Table 4).

**Table 4:** Correction grid for the BIS-15 total score, according to sex and education

Sex	Education	
	Low education (0–13 years)	High education (> 13 years)
Male	0.57	2.34
Female	− 1.61	0.08

Formula for exact direct calculation. Note that the variable education was coded as years of schooling and the variable sex was coded as males = 0 and females = 1. Adjusted BIS-15 score = raw BIS-15 score − [− 0.374 × (Education − 15.2865)] − [2.207 × (Sex – 0.7154)]

A cutoff value (37.39) was fixed at the non-parametric inner tolerance limit calculated on the 95th centile in order to obtain the maximum sensitivity.

## Discussion

The present study aimed at evaluating the psychometric properties of the BIS and providing the first normative data for the Italian general population.

Indeed, the Italian translation and adaption of the BIS by Fossati and colleagues [10] were performed on a sample consisting of college undergraduate students, whereas our sample consisted of individuals covering wider ranges of age and years of schooling.

Although the scale demonstrated acceptable internal consistency, we found that several items provided an unsatisfactory level of information about the latent trait (impulsiveness). Therefore, we developed an ad hoc checklist (see Supplementary Material 1) consisting of three statistical criteria (i.e., item-total, corrected item-total correlations, and IRT) to identify which items deserved to be removed based on internal consistency and measurement precision.

Then, we proposed a shortened version, labeled as BIS-15 (15 items; see Supplementary Material 4), that showed a satisfactory internal consistency and an almost identical measurement precision of the construct compared to the original scale.

The internal consistency of the BIS-15 via Cronbach’s alpha (0.793) was in line with previous studies investigating the psychometric properties of the BIS-11 (see review [24]) and overlapped with the Cronbach’s alpha (0.79) revealed by Fossati and collaborators [10]. Moreover, as expected, the BIS-15 score was strongly correlated with the Positive Urgency subscale of the S-UPPS-P and weakly to moderately associated with PHQ-9 and GAD-7 scores, thus demonstrating good convergent and divergent validity.

The factor analysis identified a clear four-factor solution for the BIS-15. Factor 1 included items reflecting a “pure impulsivity” (e.g., acting “on impulse,” saying and doing things without thinking, relying on luck); factor 2 consisted of items relying on planning and thinking such as planning tasks and carefully thinking; factor 3 included items reflecting difficulties in attention and concentration (e.g., difficulty in paying attention and maintaining concentration); and factor 4 consisted of items describing money troubles due to impulsivity such as difficulties in saving money and impulsive buying.

The factorial structure of the BIS-15 differed from the one proposed for the BIS-11 by Patton and colleagues [9] and partially replicated by Fossati and collaborators [10]. Indeed, our factors about planning and thinking capabilities and attention and concentration difficulties seem to replicate the factors about Attentional and Nonplanning Impulsiveness of the BIS-11. Nevertheless, we identified a “pure impulsivity” factor reflecting the tendency to act without thinking about the consequences and an “impulsive buying” factor that reflects the urgency to buy goods and spend money without planning in advance.

We found that the raw BIS-15 score was affected by formal education and sex but not by age. These results support the hypothesis of a relationship between education and impulsivity since educational attainment plays a crucial role in developing individuals’ ability to plan and foresee the consequences of their own behaviors [25]. Conversely, the relationship between impulsivity and sex is still debated because of the multi-dimensional nature of the construct that is reflected in its operationalization and thus in the heterogeneity of the assessment methods (see review [26]). 

In conclusion, the BIS-15 shows good psychometric properties and maintains the same precision as the original scale; furthermore, due to its short administration time, it could be employed by clinicians as a quicker screening tool to assess impulsivity in clinical populations (cutoff score: 37.39), such as individuals affected by psychiatric or neurological diseases, in order to address tailored pharmacological and non-pharmacological rehabilitation interventions.

## Supplementary Information

Below is the link to the electronic supplementary material.

Supplementary file1 (DOCX 19 KB)

Supplementary file2 (DOCX 20 KB)

Supplementary file3 (PNG 65 KB)

Supplementary file4 (DOCX 23 KB)
