# Supplementary material for: Validation of a short Italian version of the Barratt Impulsiveness Scale (BIS-15) in non-clinical subjects: psychometric properties and normative data
Source: Neurol Sci. 2022 Apr 11;43(8):4719–27. doi: 10.1007/s10072-022-06047-2 (PMC9349262; doi:10.1007/s10072-022-06047-2)
Supplement: Supplementary file 1 — Supplementary file1 (DOCX 19 KB) [file 10072_2022_6047_MOESM1_ESM.docx]

|  | Criteria | | |  |
| --- | --- | --- | --- | --- |
| Items | Item-total correlation | Corrected item-total correlation | Information (IRT) | Outcome |
| Item 1 | 🗸 | 🗸 | 🗴 | Included |
| Item 2 | 🗸 | 🗸 | 🗸 | Included |
| Item 3 | 🗴 | 🗴 | 🗴 | Excluded |
| Item 4 | 🗸 | 🗸 | 🗸 | Included |
| Item 5 | 🗸 | 🗸 | 🗸 | Included |
| Item 6 | 🗴 | 🗴 | 🗴 | Excluded |
| Item 7 | 🗴 | 🗴 | 🗴 | Excluded |
| Item 8 | 🗸 | 🗸 | 🗸 | Included |
| Item 9 | 🗸 | 🗸 | 🗸 | Included |
| Item 10 | 🗸 | 🗸 | 🗴 | Included |
| Item 11 | 🗸 | 🗴 | 🗴 | Excluded |
| Item 12 | 🗸 | 🗸 | 🗴 | Included |
| Item 13 | 🗸 | 🗸 | 🗴 | Included |
| Item 14 | 🗸 | 🗸 | 🗸 | Included |
| Item 15 | 🗴 | 🗴 | 🗴 | Excluded |
| Item 16 | 🗴 | 🗴 | 🗴 | Excluded |
| Item 17 | 🗸 | 🗸 | 🗸 | Included |
| Item 18 | 🗸 | 🗸 | 🗴 | Included |
| Item 19 | 🗸 | 🗸 | 🗸 | Included |
| Item 20 | 🗸 | 🗴 | 🗴 | Excluded |
| Item 21 | 🗴 | 🗴 | 🗴 | Excluded |
| Item 22 | 🗸 | 🗸 | 🗸 | Included |
| Item 23 | 🗴 | 🗴 | 🗴 | Excluded |
| Item 24 | 🗸 | 🗴 | 🗴 | Excluded |
| Item 25 | 🗸 | 🗸 | 🗸 | Included |
| Item 26 | 🗸 | 🗴 | 🗴 | Excluded |
| Item 27 | 🗸 | 🗴 | 🗴 | Excluded |
| Item 28 | 🗸 | 🗴 | 🗴 | Excluded |
| Item 29 | 🗴 | 🗴 | 🗴 | Excluded |
| Item 30 | 🗸 | 🗴 | 🗴 | Excluded |

**Supplementary Material 1. Outcome checklist.**

Note: 🗸, Satisfied; 🗴, Unsatisfied
