# Supplementary material for: Validation of a short Italian version of the Barratt Impulsiveness Scale (BIS-15) in non-clinical subjects: psychometric properties and normative data
Source: Neurol Sci. 2022 Apr 11;43(8):4719–27. doi: 10.1007/s10072-022-06047-2 (PMC9349262; doi:10.1007/s10072-022-06047-2)
Supplement: Supplementary file 2 — Supplementary file2 (DOCX 20 KB) [file 10072_2022_6047_MOESM2_ESM.docx]

**Supplementary Material 2. Difficulty (β) and discrimination (α) parameters for each of the 30 BIS items.**

| Items | β1 | β2 | β3 | α |
| --- | --- | --- | --- | --- |
| Item 1 | -1.852*** | 1.350*** | 5.476*** | 0.596*** |
| Item 2 | -0.428*** | 1.607*** | 3.404*** | 1.636*** |
| Item 3 | -70.61 | 18.731 | 98.214 | 0.023 |
| Item 4 | -0.048 | 2.502*** | 5.524*** | 0.988*** |
| Item 5 | -0.325** | 2.018*** | 3.942*** | 0.962*** |
| Item 6 | -4.802*** | -1.205*** | 2.208*** | 0.480*** |
| Item 7 | -211.404 | 13.822 | 287.564 | 0.005 |
| Item 8 | -0.862*** | 1.359*** | 3.809*** | 1.055*** |
| Item 9 | -1.756*** | 0.975*** | 3.566*** | 0.93*** |
| Item 10 | -1.795*** | 0.626*** | 3.181*** | 0.771*** |
| Item 11 | 0.006 | 2.167*** | 4.339*** | 0.632*** |
| Item 12 | -1.876*** | 2.634*** | 10.001*** | 0.457 |
| Item 13 | -1.98*** | 1.437*** | 4.629*** | 0.547*** |
| Item 14 | -0.201** | 1.595*** | 3.207*** | 1.745*** |
| Item 15 | 19.73 | -0.861 | -21.053 | -0.752 |
| Item 16 | 3.238 | 10.556 | 22.614 | 0.248 |
| Item 17 | -0.930*** | 1.079*** | 2.648*** | 1.707*** |
| Item 18 | -0.544*** | 2.317*** | 4.565*** | 0.804*** |
| Item 19 | -0.736*** | 1.079*** | 2.554*** | 1.830*** |
| Item 20 | -4.282** | 3.286** | 11.648** | 0.247** |
| Item 21 | 4.378** | 11.404* | 17.247* | 0.302* |
| Item 22 | -0.327** | 2.385*** | 4.630*** | 0.927*** |
| Item 23 | -1.870* | 7.739* | 15.248* | 0.196* |
| Item 24 | -0.301 | 3.045*** | 6.864*** | 0.587*** |
| Item 25 | 1.059*** | 3.052*** | 4.842*** | 0.908 |
| Item 26 | -0.304** | 2.300*** | 4.450*** | 0.824*** |
| Item 27 | -2.911*** | 1.864*** | 6.757*** | 0.431*** |
| Item 28 | 0.949*** | 3.410*** | 5.072*** | 0.701*** |
| Item 29 | -20.034 | -3.543 | 13.571 | 0.089 |
| Item 30 | -3.086*** | 2.395** | 9.344*** | 0.336*** |

Note: * *p*<0.05, ** *p*<0.01, *** *p*<0.001.
