# Supplementary figures and images for: Validation of a short Italian version of the Barratt Impulsiveness Scale (BIS-15) in non-clinical subjects: psychometric properties and normative data
Source: Neurol Sci. 2022 Apr 11;43(8):4719–27. doi: 10.1007/s10072-022-06047-2 (PMC9349262; doi:10.1007/s10072-022-06047-2)

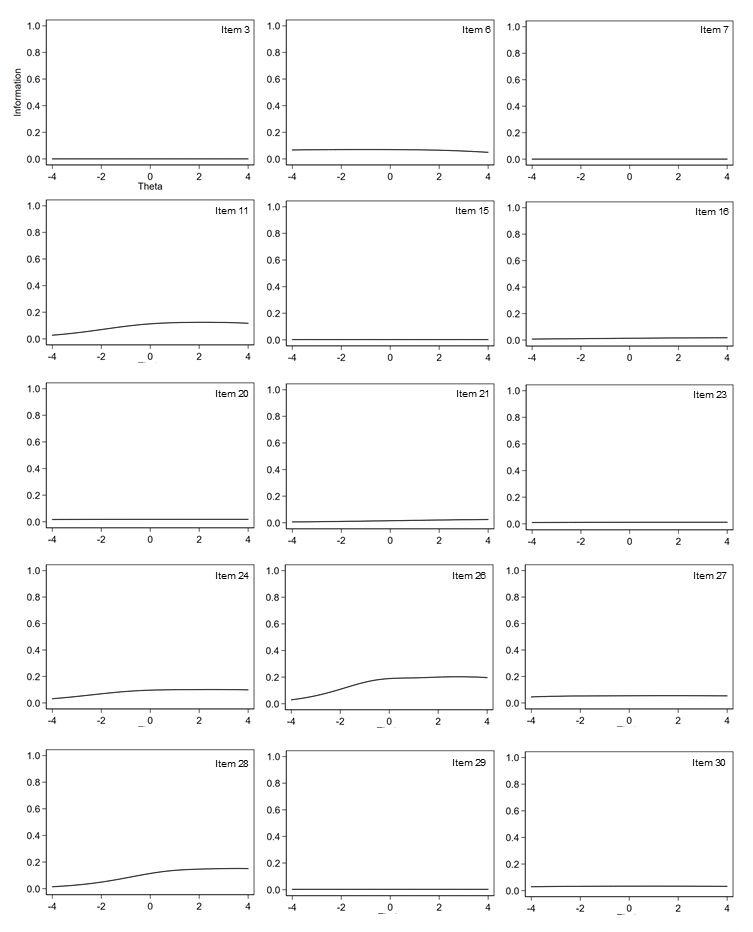

Supplement: Supplementary file 3 — Supplementary file3 (PNG 65 KB) [file 10072_2022_6047_MOESM3_ESM.png]
