# Supplementary material for: Validation of a short Italian version of the Barratt Impulsiveness Scale (BIS-15) in non-clinical subjects: psychometric properties and normative data
Source: Neurol Sci. 2022 Apr 11;43(8):4719–27. doi: 10.1007/s10072-022-06047-2 (PMC9349262; doi:10.1007/s10072-022-06047-2)
Supplement: Supplementary file 4 — Supplementary file4 (DOCX 23 KB) [file 10072_2022_6047_MOESM4_ESM.docx]

**BIS-15 (Italian short-version)^1,2^**

Gentile Signore/a, nel seguente questionario sono elencate una serie di situazione nelle quali le persone usualmente vengono a trovarsi nel corso della propria vita. Ad ogni frase può rispondere scegliendo la modalità che si presta meglio a descriverLa. Il questionario va compilato nella sua totalità secondo quanto Lei pensa e senza l’aiuto di altre persone. Ovviamente, non esistono risposte giuste o sbagliate; è importante solo descrivere i propri sentimenti personali.

|  | Mai/ Raramente | Talvolta | Spesso | Quasi sempre/ Sempre |
| --- | --- | --- | --- | --- |
| 1. Pianifico le attività attentamente* |  |  |  |  |
| 1. Faccio le cose senza pensarci |  |  |  |  |
| 1. Mi affido alla sorte |  |  |  |  |
| 1. Non “focalizzo l’attenzione |  |  |  |  |
| 1. Ho autocontrollo* |  |  |  |  |
| 1. Mi concentro facilmente* |  |  |  |  |
| 1. Risparmio con regolarità* |  |  |  |  |
| 1. Sono un attento pensatore* |  |  |  |  |
| 1. Faccio progetti per una sicurezza lavorativa* |  |  |  |  |
| 1. Dico cose senza pensare |  |  |  |  |
| 1. Agisco “d’impulso” |  |  |  |  |
| 1. Mi annoio facilmente quando devo risolvere dei problemi concettuali |  |  |  |  |
| 1. Agisco sull’impulso del momento |  |  |  |  |
| 1. Compro le cose d’impulso |  |  |  |  |
| 1. Spendo più di quello che guadagno |  |  |  |  |

1. Traduzione italiana curata da Andrea Fossati, Michela Donini, Deborah Donati

2. Versione ridotta realizzata da: Gianpaolo Maggi, Manuela Altieri, Ciro Rosario Ilardi, Gabriella Santangelo

**BIS-15**

**Istruzioni per la correzione**

| **Scoring** | | **Scoring item reverse*** | |
| --- | --- | --- | --- |
| • Mai/ Raramente  • Talvolta  • Spesso  • Quasi sempre/ Sempre | 1  2  3  4 | • Mai/ Raramente  • Talvolta  • Spesso  • Quasi sempre/ Sempre | 4  3  2  1 |

Item reverse sono contrassegnati con un asterisco (*)

**BIS-15 (English short-version)^1,2^**

People differ in the ways they act and think in different situations.

This is a test to measure some of the ways in which you act and think.

Read each statement and select an appropriate answer.

Do not spend too much time on any statement. Answer quickly and honestly.

|  | Never/ Rarely | Occasionally | Often | Almost Always/ Always |
| --- | --- | --- | --- | --- |
| 1. I plan tasks carefully* |  |  |  |  |
| 1. I do things without thinking |  |  |  |  |
| 1. I am happy-go-lucky |  |  |  |  |
| 1. I don’t “pay attention” |  |  |  |  |
| 1. I am self-controlled* |  |  |  |  |
| 1. I concentrate easily* |  |  |  |  |
| 1. I save regularly* |  |  |  |  |
| 1. I am a careful thinker* |  |  |  |  |
| 1. I plan for job security* |  |  |  |  |
| 1. I say things without thinking |  |  |  |  |
| 1. I act “on impulse” |  |  |  |  |
| 1. I get bored easily when solving thought problems |  |  |  |  |
| 1. I act on the spur of the moment |  |  |  |  |
| 1. I buy things on impulse |  |  |  |  |
| 1. I spend or charge more than I earn |  |  |  |  |

1. Instructions and items provided by: Jim H. Patton, Matthew S. Stanford, Ernest S. Barratt

2. Short-version proposed by: Gianpaolo Maggi, Manuela Altieri, Ciro Rosario Ilardi, Gabriella Santangelo

**BIS-15**

**Scoring instructions**

| **Scoring** | | **Scoring item reverse*** | |
| --- | --- | --- | --- |
| • Never/ Rarely  • Occasionally  • Often  • Almost Always/ Always | 1  2  3  4 | • Never/ Rarely  • Occasionally  • Often  • Almost Always/ Always | 4  3  2  1 |

Reverse item are marked by an asterisk (*)
